# Smartphone addiction and malevolent creativity—the mediating role of psychological capital and the moderating role of self-concept clarity

**DOI:** 10.3389/fpsyt.2025.1574738

**Published:** 2025-04-28

**Authors:** Wei Li, Ying Zhou

**Affiliations:** ^1^ Faculty of Education, Qufu Normal University, Qufu, Shandong, China; ^2^ Faculty of Education, Jiangxi University of Technology, Nanchang, Jiangxi, China

**Keywords:** smartphone addiction, malevolent creativity, psychological capital, self-concept clarity, mediating, moderating

## Abstract

**Purpose:**

This study examines the correlation among smartphone addiction (SA) and malevolent creativity, as well as the underlying mechanisms involved. A moderated mediation framework was established to investigate the impact of SA on malevolent creativity in college students, taking into account the mediating influence of psychological capital and the moderating effect of self-concept clarity (SCC).

**Method:**

This survey was conducted from May 10 to August 15, 2024 at a higher education institution in a central city of China. A total of 1,091 Chinese college students completed measuring SA, psychological capital, SCC, and malevolent creativity. Correlation and mediational moderation studies were performed utilizing SPSS (version 25.0) and AMOS (version 23.0).

**Results:**

The association research indicated that SA exhibited a beneficial association with malevolent creativity and SCC, while demonstrating an adverse relationship with psychological capital. Additionally, malevolent creativity had an adverse association with psychological capital and positively and a good correlation with SCC. Furthermore, psychological capital was negatively correlated with SCC. The structural equation modeling investigation indicated that SA directly predicts malevolent creativity in college students, but psychological capital exerts an indirect predictive influence between the two variables. Specifically, psychological capital was found to be a partial mediator and buffer between SA and malevolent creativity. In addition, SCC moderated the second half of the “smartphone addiction →psychological capital → malevolent creativity” path and moderated the “smartphone addiction → malevolent creativity” path.

**Conclusion:**

SA directly and indirectly affects malevolent creativity through psychological capital. SCC serves as a mitigating factor between SA and malevolent creativity. The association between psychological capital and malevolent creativity is great when the degree of self-concept is elevated, whereas the correlation between SA and malevolent creativity is also rather substantial.

## 1 Introduction

In the era of digital transformation, smartphones have become indispensable tools for social connection, entertainment, commerce, and financial management. For example, scholars believe that the diversity and ease of smartphone app usage can help adolescents to continuously search for learning resources (1). Additionally, other scholars who believe that smartphones facilitate knowledge sharing and communication between teachers, students, and peers, which can improve teamwork and the efficiency of learning (2). They also believe that smartphones create a new interactive space for activities such as online teaching and interpersonal communication, which makes adolescents’ learning easier, more convenient, and efficient (3), which has a positive effect on their academic performance and social development (4). Smartphone use facilitates people’s productivity and life, and university students benefit from it.

However, excessive use of smartphones may also cause university students to experience adverse psychological and behavioral effects, which could affect their social functioning (5). A survey found that the SA detection rate among college students ranged from 4.05% to 27.4%, and the potential addiction rate may reach up 58.33% (6). Moreover, SA can substantially hinder an individual’s personal development and social integration such as causing negative emotions (7), poor academic performance (8), social communication anxiety (9), and decreased sleep quality (10). SA also leads to serious psychiatric disorders (11). In 2015, the World Health Organization officially categorizes SA under public health priorities and pointed out an urgent need to strengthen research on factors influencing SA and the intervention process (12). Smartphone use and addiction has become a global public health concern (13).

Although smartphone addictive behaviors may negatively impact individuals, studies have revealed the relationship between SA and the ability to generate malicious ideas, termed creativity (14, 15). Creativity impacts personal progress, scientific and technological innovation, and social development, and it is usually regarded as a positive quality. However, creativity can be a double-edged sword; when it is driven by malevolence, it can significantly impact society or individuals negatively (16). malevolent creativity refers to consciously and premeditatedly harming oneself or others in novel ways (17). Research has found that malevolent creativity jeopardizes society’s security, stability, and development (18). As a special group, college students’ malevolent creativity not only affects their personal development, but may also have far-reaching effects on their group, society, and future career (19). Therefore, it is socially beneficial to study the influence mechanisms underlying SA that drive college students’ malevolent creative behaviors and investigate efficient moderation techniques to mitigate the potential risks posed by malevolent innovation to students, educational institutions, and society at large.

SA is intimately linked to the behavior of an individual and may influence an individual’s psychological capital; however, limited studies have been conducted on the mediation role of psychological capital in the conversion of smartphone addicted behaviors into malevolent creativity (20). Research indicates that the connection between behavioral addiction and creativity may be affected by basic psychological factors, such as SCC (21). SCC has been a mediator and moderating feature in research connected to SA (22, 23). Nevertheless, there exists a scarcity of studies about the correlation between psychological capital and SCC, as well as SA and malevolent creativity. This led the authors to consider the impact of SA on individuals’ malevolent creativity and the potential influence of psychological capital and SCC in this dynamic. The link between psychological capital, SCC, SA, and malevolent creativity remains inadequately explored. Consequently, additional research may be pertinent to the mental health, academic performance, and career advancement of college students.

The study seeks for investigate the correlation between SA and malevolent creativity in undergraduates. while also assessing the mediating role of psychological capital and the moderating effect of SCC within the framework. The subsequent sections will examine these four factors and their interrelationships.

## 2 Literature review and hypotheses

### 2.1 Smartphone addiction

Before the smartphones emerge, several research concentrated on the topic of addiction to classic non-smartphones (24). Smartphones have largely replaced traditional cell phones (25). Most scholars now regard non-smartphones addiction and Smartphones addiction as the same concept (26). Researchers have defined SA on different levels. For example, on the psychological level, SA refers to an individual’s strong psychological dependence on the phone, desire for uninterrupted use of the phone, and extreme anxiety and inner pain when separated from the phone (27). SA behaviorally denotes an individual’s inappropriate and excessive utilization of the device, resulting in substantial adverse effects on both the individual and society (26). In clinical psychology, SA is characterized as a psychological dependence on excessive smartphone use, resulting in diminished self-control, impaired social functioning, and the emergence of psychological or behavioral issues (28). While experts have not achieved unanimity on the definition of SA, they concur that it encompasses three characteristics (1): uncontrollable use of the phone; always paying attention to new information on the phone and ignoring the immediate environment (29) (2); the emergence of a psychological withdrawal reaction, e.g., anxiety when the individual cannot use the smartphone, producing adverse emotions such as short- or long-term anxiety, irritability, and depression (30); and (3) SA can negatively affect an individual’s academics, health, and other aspects (31).

SA is characterized by an individual’s compulsive and excessive utilization of a smartphone, creating a psychological dependence and a loss of self-control regarding the use of smartphone. This may eventually affects their social functioning and produces psychological or behavioral problems (32). This concept has been extensively utilized in the domain of higher learning, sociology, and psychology (33, 34). According to the existing literature, SA is multidimensional and complex (35). Scholars have generated different perspectives for analyzing its inner essentials (36). The renowned scale is the SA scale created by Bianchi and Phillips, a 17-item scale used to quantify the sense of place from low to high on four dimensions: loss of control, withdrawal, avoidance, and ineffectiveness (28). Studies have shown that cell phone dependence triggers impaired physiological, psychological, behavioral, and social functioning of individuals and causes new types of social problems (37). SA results in physical health issues, including diminished sleep quality (38), impaired vision (39), and whiplash (40), with psychological disorders such as anxiety, sadness, and suicide thinking (41, 42). SA also affects people’s interpersonal relationships (43) and academic performance (44). Smartphone-addicted college students show antisocial tendencies and are prone to engage in criminal behavior (45) and many other negative behaviors.

### 2.2 Malevolent creativity

Guilford first suggested that “creativity is an inherent ability in all individuals and constitutes a facet of intelligence.” (46, 47). Academics have not achieved unanimity on the definition of creativity owing to its intricacy. Researchers have provided several views of creativity throughout different periods and circumstances (48). The definitions of creativity reveals that each researcher’s orientation is consistent with their adopted product, process, personality, and environment. This study defines creativity as the capacity of an individual to generate unique (original and unforeseen) and appropriate (contextually relevant) ideas and products within a certain setting (49).

Creativity has always seemed “good for the individual, good for society.” However, creativity can also have a negative impact on individuals and society if it is used malevolently, i.e. creativity has a “dark side” (16). Malevolent creativity stands for a common expression of the “dark side” of creativity that deliberately inflicts harm on individuals, property, processes, symbols, and more (50). Both malevolent and general creativity require that individuals generate ingenious and useful ideas or solutions. According to research, general creativity predicts malevolent creativity (17). Malevolent creativity suggests that an individual’s motivation is to intentionally harm other objects. Thus, it possesses some characteristics of traditional creativity such as novelty and effectiveness. However, malevolent creativity possesses its unique characteristics—purposefulness and harmfulness (51).

SA has a multidimensional and complex structure that may impair the physiological, psychological, behavioral, and social functioning of individuals. Research regarding malevolent creativity has been largely conducted under the theoretical framework of traditional creativity research. The first and most famous theoretical framework of creativity is the creativity “four elements model” (4Ps), which posits that creativity can be described from the four elements of creativity: people, media, process, and product. Amabile’s proposed model of creativity comprises cognitive, individual, motivational, and social elements, encompassing domain-specific abilities, creativity-related skills, and task motivation (52).

Sekowski added “social environment factors” to the model (53). The creativity investment theory suggests that intelligence, knowledge, cognitive style, personal characteristics, motivation, and the environment all have an impact on creativity, and that creativity is the result of the interaction of individual mental processes and environmental factors (54). Furthermore, in the onset of the 21st century, Plucker, Beghetto, and Dow posited that creativity arises from the interplay of abilities, processes, and the environment, enabling a person or group to generate innovative and valuable output (50). Kaufman and Beghetto offered a model for creativity development, asserting that creativity is not solely an individual’s autonomous capability but is also significantly influenced by contextual factors, the nature of the task, and the characteristics of the problem (55). The 5A framework introduced by Glaveanu et al. is a multifaceted theory of creativity aimed at offering an extensive comprehension of the processes and expressions of creativity (56). It suggests that five elements—actor, action, product, audience, and availability—influence creativity and emphasizes the socio-cultural context and the interaction between the individual and the environment more than the 4P framework proposed by Rhodes (57). The fourth concept is the dark triad theory, which posits that narcissism, Machiavellianism, and psychopathy may predispose individuals to employ creativity for deceit, manipulation, or other nefarious actions (58).

All previously discussed theories are relevant to the analysis of the relationship between SA and creativity. The investment theory of ingenuity and the modeling model of malevolent creativity are particularly relevant to this study, providing the theoretical basis for investigating the connection between SA and malevolent creativity.

Literature shows that researchers have mostly concentrated on the correlation between SA and creativity (59), whereas less attention has been devoted to the processes through which SA influences malevolent creativity. In line with the theories above, several recent studies have discussed the factors of malevolent creativity such as intelligence, knowledge, thinking styles, personal traits, motivation, and the environment (51). These studies explain the processes involved in malevolent creativity. Numerous recent research have investigated the correlation between malevolent creativity and the factors of environment (60), perception (61), and emotion (62).Presently, limited research exists on SA and harmful creativity; some studies have identified an association between SA and creativity (63). Additional research have documented the correlation between SA and IQ, knowledge, cognitive styles, personal characteristics, motivation, and environmental factors. For example, scholars have argued that smartphone-addicted individuals are often exposed to virtual environments, which may contain malevolent content such as cyber-violence and undesirable information, which blur the boundaries between moral and immoral behaviors and distort their values and beliefs (64). Some research reports also conclude that SA can trigger aggressive behavior and deviant psychology, both of which reveal malevolent tendencies in creative problem solving (65). A substantial amount of data indicates that college students’ inclination toward SA correlates with relationship distress (43), loneliness (66), low subjective well-being (67), depression (68), and psychological anxiety (69), which are all significantly positively correlated; they are all significant factors that trigger malevolent creativity. Therefore, this study suggests that pathological smartphone behavior may significantly produce malevolent creativity among college students. We put out the subsequent hypothesis:

H1: SA is positively associated with malevolent creativity.

### 2.3 Psychological capital

Goldsmith first proposed the concept of psychological capital in 1997. “Psychological capital is a combination of an individual’s beliefs, attitudes, and perceptions about work, ethics, and self-life, and is a personality trait that significantly increases the level of motivation and creativity among employees (70).” Subsequently, psychological capital has received extensive attention from positive psychologists and positive organizational behaviorists. In 2004, Luthans et al. (71, 72) defined psychological capital as an individual’s “positive psychological strengths” and believed that it includes characteristics that can affect an individual’s positive conduct. Capital is the psychological condition that can affect a person’s constructive behavior, characterized externally by the amalgamation of optimism, hope, resilience, and self-efficacy experienced by individual employees. The degree of psychological capital exerts a substantial beneficial impact on attitudes and behaviors (73). Numerous theories exist on psychological capital. For example, Letcher (74) argues that psychological capital captures the personality traits that influence an individual’s performance, including openness, stability, and agreeableness. In addition, Avolio et al. based on the state theory, argued that psychological capital is a combination of positive psychological states that enhance an individual’s performance and happiness at work, and that these positive psychological states produce positive organizational behaviors that can be invested in and developed toward a higher job performance and satisfaction (75). In 2004, Luthans analyzed and compared psychological capital, human capital, and social capital and concluded that psychological capital is a positive psychological state that can be invested in and developed like human and social capital to generate positive competitiveness (71). Although scholars hold different understandings of the connotations of psychological capital, they all view psychological capital from a positive psychology perspective as quality characteristics that can stimulate an individual’s potential to produce positive results (76). This study characterizes mental assets as a favorable psychological condition exhibited by an individual during growth and development, which is a crucial psychological resource for enhancing individual advancement (77).

Although various scholars interpret psychological capital differently, measuring psychological capital and its characteristics is mature and stable; self-efficacy, optimism, hope, and resilience are the widely recognized structural dimensions of psychological capital (78). These four promote each other and work synergistically to positively influence mindset, behavior, among others (79). Some studies indicate that psychological capital has become a key factor which can reduce the probability of SA (80); others have shown that the four core components of psychological capital—self-efficacy, optimism, resilience, and hope—are all linearly and negatively related to SA (81–83). Positive psychology also suggests that there are two forces—positive and negative—that oppose each other in a person’s inner world. If the positive force is stimulated and enhanced, the negative force will be suppressed or eliminated (84). Therefore, psychological capital as a positive force can inhibit and eliminate the negative effects of SA. Scholars have found that when the mediating variable, psychological capital, is added to SA and mental health, the regression coefficient between SA and mental health decreases significantly. The rate and degree of SA among undergraduates with higher psychological capital is significantly lower (85). Other scholars demonstrated that et al. conducted a study on undergraduate nursing students, revealing that self-control partially mediated the positive correlation between stress perception and the propensity for SA. Furthermore, psychological capital could influence the relationship between self-control and SA propensity (86). Consequently, we bring out one more hypothesis:

H2a: Psychological capital is negatively related to SA.

Psychological capital is a constructive psychological energy and intrinsic resource that individuals gradually cultivate during their growth and development (76). Researchers have determined that psychological capital exerts a substantial positive influence on innovative work behavior (87). Psychological capital exerts a substantial moderating and mediating influence on the relationship between job stress and creativity (88). However, more studies have argued for a relationship with malevolent creativity based on the components of psychological capital such as optimism, mental toughness, and hope (19). It has also been found that mental toughness protects people from stressful environments, which reduces cognitive malice and encourages an optimistic approach to problem solving (19). Mental toughness has an adverse relationship with aggressive creative behavior. Some research have identified an association between self-efficacy and malevolent creativity, indicating that persons with elevated self-efficacy are more inclined to evade or disengage from criminal and other antisocial behaviors (89). Some findings show that hope promotes positive emotions in individuals, enables individuals to use positive psychological resources to cope with setbacks, reduces psychological problems in individuals, and is essential for the prevention and treatment of disruptive behaviors (90). Research indicates that the elements of psychological capital influence malevolent creativity. The synergistic effect of psychological capital elements exerts a more substantial influence than any one factor alone (91). Thus, psychological capital can positively predict malevolent creativity. It culminates in the subsequent hypothesis:

H2b: Psychological capital negatively predicts malevolent creativity.

As mentioned above, SA negatively predicts college students’ psychological capital (92). Individuals with low psychological capital display more problematic and aggressive behaviors (93), which increases malevolent creativity (94). Hence, it can be posited that SA influences malevolent creativity by diminishing psychological capital and augmenting malevolent creativity. Thus, the following hypotheses are proposed:

H2: Psychological capital mediates and buffers the relationship between SA and malevolent creativity.

### 2.4 Self-concept clarity

SCC is connected to the clarity, beliefs, internal consistency, and consistency of an individual’s self-perception (95). This significant personal resource aids individuals in elucidating their objectives (96), facilitating effective self-management, fostering a profound sense of purpose (97), enhancing overall well-being (98), reducing negative emotions (99), and promoting personal development while ameliorating maladaptive behaviors (100). Research indicates that persons possessing high SCC are less prone to exhibit SA behaviors (101). Accordingly, a correlation exists between SCC and SA. In view of this, we proposed this hypothesis:

H3a: SCC is correlated with SA.

Malevolent creativity is an advanced cognitive ability influenced by a combination of complex factors. Research has emphasized that an individual’s personality traits influence malevolent creativity through maliciousness (102). Self-concept is a crucial element of personality structure, encompassing self-assessments of competence, self-esteem, and social duties (103). Individuals with elevated SCC exhibit enhanced self-regulation and a superior capacity to withstand the impact of adverse external information (104), as well as a more objective and favorable self-evaluation to satisfy their own standards (96). Such groupings exhibit a low tendency for conflict or animosity. Individuals possessing strong SCC exhibit greater social engagement and are more inclined to establish stable emotional connections with others compared to those with low SCC (105). These groups are more inclined to possess elevated levels of social support and resources, which is strongly inversely connected with a reduced moral sensibility and a deficiency of empathy (106). Individuals with greater SCC are said to be more active, demonstrate enhanced cooperative problem-solving activities, and exhibit reduced hostility (107). Additionally, there is research indicating that SCC reduces individuals’ negative experiences, including anxiety and depressive moods and perceptions of stress (108). Numerous studies have suggested that individuals with traits such as aggressive tendencies (94), high risk-taking, low morality (109), and non-empathy (110) may have a higher propensity for malevolent creativity. Thus, we concluded that SCC substantially affects malevolent inventiveness and proposed the research hypothesis as following:

H3b: SCC is correlated with malevolent creativity.

Investigations into the relationship between SCC and psychological capital are scarce. Multiple studies have researched the hyperlink among SCC and psychological resilience, hope, confidence, and optimism. Research on athletes indicates that SCC significantly correlates with mental toughness and influences burnout levels in athletes via mental toughness (111). A study of 2,792 adolescents and young adults aged between 11 and 24 revealed that family cohesion may have a mediating effect on subjective well-being through the “self-concept clarity-hope” link, where hope was significantly impacted by SCC. In this process, SCC has a significant effect on hope (112). Cross-lagged panel (regression) modeling has revealed a reciprocal impact link between achievement and self-concept (113). SCC may act as a favorable predictor of an individual’s psychological capital, which comprises self-efficacy, hope, optimism, and mental toughness as its four essential elements. Accordingly, the following hypothesis has been proposed:

H3c: SCC is correlated with psychological capital.

In summary, Multiple studies have indicated that the connection between psychological capital and SA is mediated or regulated by SCC. An individual’s psychological capital may also be influenced by their SCC and, thus, malign creativity. According to the literature and our preceding hypotheses, we proposed the hypotheses as follow:

H4: SCC may moderate the relationship between SA and psychological capital.

H5: SCC may moderate the relationship between SA and malevolent creativity.

**Figure 1:**
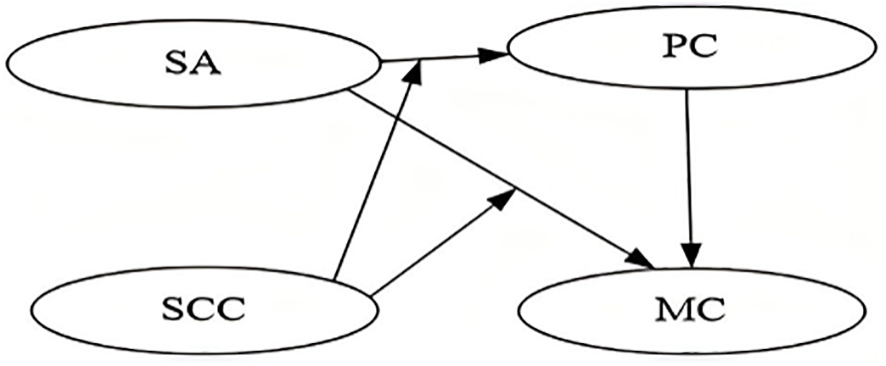
Measurement model. Factor loadings are standardized results (1180). PC, Psychological Capital; SA, Smartphone Addiction; MC, Malevolent Creativity.

In brief, there are four variables were included in the theoretical model mentioned. We hypothesized possible pathways to establish a comprehensive understanding of Smartphone Addiction, Psychological Capital, Self-Concept Clarity influencing Malevolent Creativity (see **Figure 1**).

## 3 Methodology

### 3.1 Research design

In order to reveal the relationship between smartphone addiction and malevolent creativity and the mediating role of psychological capital and the moderating role of self-concept clarity, the researchers conducted the following research design. Firstly, the connection between smartphone addiction and malevolent creativity was explored from the literature through a rooted theory approach. At the same time, the possibility of the mediating role of psychological capital and the moderating role of self-concept clarity was explored through exploratory focus interviews, and then evidence from the literature was searched to derive relevant hypotheses. Secondly, a minimum sample size was calculated based on the diversity of the students’ disciplines and the total number of questions in the questionnaire, and then the appropriate number was obtained based on stratified random sampling. Meanwhile, the questionnaires were submitted to the ethical review organization for review. Again, according to the rules of questionnaire distribution, the subjects were first informed of the motive of the survey, and then they were asked to fill out the informed consent form, and those who did not agree could withdraw from the survey. Finally, data verification and modeling were performed.

### 3.2 Participants and procedure

This investigation was undertaken in a higher education institution in central China. The college has 15 faculties with 56 undergraduate programs, 40 specialized programs, and more than 40,000 full-time enrolled students; 26 students participated in exploratory focus group interviews before completing questionnaires. This enhanced our comprehension of the behavior characteristics and psychological states of potential respondents. Most of the respondents indicated that they could not detach themselves from their cell phones. Thus, cell phones significantly influenced their life and academic pursuits. Due to the more obvious differences in the disciplinary background and daily life status of students in different colleges. In order to control for potential differences between colleges and improve the universality of the survey results, we utilized stratified random sampling method, in which all students were randomly picked by department. From May 10 to August 15, 2024, 1,200 students completed an online questionnaire.

Questionnaires with the same (consistently repeated) or missing answers were not valid, leaving 1,091 valid questionnaires. In structural equation modeling, 10 to 20 samples are required for each observed variable (114), besides basic personal information, the questionnaire had 59 items, i.e., 59 observed variables. Thus, the reasonable sample size should be 590 to 1,180. Therefore, the number of valid questionnaires collected met the requirements of structural equation modeling.

In the sample, males accounted for 35.5% and females for 64.5%; 19.4% were students under 18 years old, 36.9% were 19 years old; 20.3% were 20, 12.3% were 21, and 11.1% were 22. The freshmen accounted for 44.9%, sophomores for 22.6%, and juniors for 26.3%. Because most of the senior students were doing off-campus internships, they only accounted for 6.3%. Students majoring in humanities and social sciences accounted for 13.7%, economics and management students for 33.8%, science and engineering students for 32.4%, medical students for 5.6%, arts students for 10.4%, and others for 5.6%. In summary, the research sample was well represented.

### 3.3 Instruments

The demographic information collected included gender and specialty. The four scales were the SA, malevolent creation, psychological capital, and SCC scales. The English portions were translated into Chinese and tested using reverse translation to resolve inconsistencies prior to data collection. The scale selected for SA was established by Louis Leung et al. (115) Derived from the Bianchi and Phillips problematic cell phone use scale, a 17-item scale categorized into four dimensions: loss of control, withdrawal, avoidance, and ineffectiveness.

The SA scale is a set of self-assessment statements regarding addictive behaviors, withdrawal behaviors, negative affect, and social comfort, which require adult responses. Children, adolescents, and older adults have all been included in its scope of application. The items are assessed utilizing a 5-point Likert scale. The content consistency coefficient of the scale was 0.951.

We used the malevolent creativity scale developed by Hao Ning (116) and others to assess the level of malevolent creativity behaviors exhibited by college students. It consists of 13 items classified into three dimensions: hurting others, lying, and trickery. The frequency of malevolent thoughts in everyday life was measured using a 5-point Likert scale; a higher overall score indicated a higher level of hostile innovation in daily life. The internal reliability coefficient for this level was 0.974.

The psychological capital scale developed by Luthans, Avolio, and Avey (72) was used. It comprises four dimensions: self-efficacy, hope, optimism, and resilience. The original scale consisted of 24 questions with three reverse questions, which were duplicated with other related items in terms of the meaning for a more concise test. For more conciseness, these three questions were deleted; the final scale had 18 items. The study found that the positive psychological qualifications had a content consistency coefficient of 0.980.

This study used the SCC scale created by Campbell et al. (95), which comprised of 12 questions; questions 6 and 11 were reverse scored. A 5-point Likert scale was employed, with elevated scores signifying greater SCC. The internal consistency coefficient of the SCC scale was 0.946.

### 3.4 Data analysis

SPSS (version 25.0) and AMOS (version 23.0) were used in this study to process and analyze the data. Because this study’s reliance on self-assessment questionnaires for data collection may introduce typical method bias into the results. Therefore, in order to keep the validity of data analysis, prior to data processing, a Harman’s one-way test for common method bias was employed (117).

The findings demonstrated that each of the six factors’ eigenvalues was greater than 1, and all the six factors had contribute 73.31% to the total variance, with the first factor’s total variance being 36.73%, which was lower than the critical 40%, indicating that the data did not exhibit a significant common method bias effect. Subsequently, the evaluation of the data was performed via descriptive statistics, correlation evaluation, and validation of models based on the research hypotheses. The data’s centralized and discrete trends were analyzed using descriptive statistical analysis. Pearson correlation coefficients were computed to examine the correlations among independent, mediating, dependent, and moderating variables. Based on the correlations, AMOS 24.0 was utilized to further validate the stated research hypotheses and examine the model’s mediating and moderating roles.

## 4 Results

### 4.1 Descriptive statistics and correlation analysis

Measures of central tendency, dispersion values, and Pearson matrix correlation coefficients for mobile phone addiction malevolent creativity, psychological capital, and SCC Were worked out by means of SPSS 25.0. The results of the descriptive statistical evaluation are presented in **Table 1**, while the Pearson matrices coefficients of association are displayed in **Table 2**.

**Table 1:** Descriptive statistical analysis.

	M	SD
Malevolent Creativity	1.7364	.79390
Smartphone Addiction	2.3187	.82563
Psychological Capital	3.6351	.80442
Self-Concept Clarity	2.4150	.86474

**Table 2:** Correlation of the 4 observed variables.

	M	SD	SA	MC	PC	SCC
SA	2.319	0.826	1			
MC	1.736	0.794	.444**	1		
PC	3.635	0.804	-.279**	-.229**	1	
SCC	2.415	0.865	.738**	.410**	-.279**	1

**p<0.01.

As shown in **Table 2**, The method of Pearson correlation analysis was applied to assess the relationships within the elements in the framework. All variables were correlated. Students’ malevolent creativity revealed a positive statistical related to SA (r=0.444, p<0.01) and self-concept (r=-0.410, p<0.01), yet negatively associated with psychological capital (r=-0.229, p<0.01); SA shown an inverse association among psychological capital (r=-0.279, p<0.01), positively related to SCC (r= 0.738, p<0.01) exhibited positively associated; mental assets exhibited an adverse statistical association with SCC (r=-0.279, p<0.01). The outcomes of the correlation analyses furnish early support for the later tests of mediating and moderating effects.

### 4.2 Structural equation analysis

#### 4.2.1 Smartphone addiction and malevolent creativity

The study constructed a structural equation model of SA and malevolent creativity, and by fitting the model, it was found that: the CMIN/DF was 2.987, which was within the standard interval of 1-3; the RMSEA was 0.043, which was less than 0.08; and the figures of the TLI, CFI, RFI, and NFI were 0.960, 0.965, 0.936, and 0.944, correspondingly, which were all greater than 0.9, which meets the standard. Thus, the model fit was good. Contingent upon the data outcomes evaluation, the path factor of SA and malevolent creativity was 0.514, and the P-value was less than 0.001, reaching the significant level.

#### 4.2.2 The mediating function of psychological capital

The Bootstrap was utilized in AMOS for the analysis of what is the mediation impact of mental assets. The indicators of the model’s fitting quality regarding the mediating effect of psychological capital met the requirements: the CMIN/DF was 2.546, which was within the standard interval of 1-3; the RMSEA was 0.038, which was less than 0.08, and the figures of the TLI, CFI, RFI, and NFI were 0.967, 0.972, 0.948, and 0.956 respectively, which were all greater than 0.9 and met the standard. The model fit was good. According to **Table 3**, the absolute values of all path coefficients are between 0 and 1, and the P-values are less than 0.001, reaching the significant level. The coefficient of the path associated with SA malevolent creativity is 0.480. Meanwhile, the coefficient of the path associated with SA and psychological capital is -0.325, and the hypothesis H2aholds. Thus, the stronger the SA, the lower the degree of psychological capital; the inferior the SA, the superior the degree of psychological capital.

**Table 3:** Results of hypothesis testing for direct effects.

hypothetical	Estimate	S.E.	C.R.	P
SA→MC	0.482	0.037	13.667	***
PC→MC	-0.079	0.03	-2.719	0.007
SA→PC	-0.324	0.034	-9.76	***

***p<0.000.

The Bootstrap approach was employed to examine mediating effects. Drawing on Hayes’ study, the study set the sample size for Bootstrap sampling to 2,000 and used the percentile and bias correction methods to find 95% confidence intervals for the mediating effect (118). The findings indicated that the indirect impact estimate of “smartphone addiction→psychological capital→malevolent creativity” was 0.026, P=0.044<0.05, indicating that psychological capital functioned as a middleman between SA and malevolent creativity; the direct impact estimate was 0.482, P=0.01<0.05, as represented in **Table 4**.

**Table 4:** Results of the test of the mediating role of psychological capital.

Independent Variable	Dependent Variable	Intermediary Variable	Direct Effect	Indirect Effect	Total Effect
SA	MC		0.482 (P=0.01<0.05)		0.508 (P=0.00<0.05)
SA	MC	PC		0.026 (P=0.044<0.05)	

#### 4.2.3 The moderating role of SCC

We tested the decreasing impact of SCC by constructing interaction items. The SA scale had 17 items, malevolent creativity had 13, psychological capital had 18, and SCC had 11. In this study, the items of each scale were packed for averaging and the interaction term was calculated. Secondly, the SCC moderating role model was plotted in AMOS based on the calculation results.

The SCC regulation model fit indicators met the requirements, CMIN/DF was 2.969 and was within the standard interval of 1-3; RMSEA was 0.043, less than 0.08; and the values of TLI, CFI, RFI, and NFI were 0.949, 0.956, 0.925, and 0.936, which are greater than 0.9, respectively, demonstrating that the model is well-suited. According to **Table 5**, The findings indicated that The path from SCC×SA to PC has a positive estimate of 0.127 (P=.0000), suggesting that the interaction of self-concept clarity and cell phone addiction has a significant positive effect on positive self-efficacy. The path from SCC×SA to MC has a positive estimate of 0.117 (P=.0000), suggesting that the interaction of self-concept clarity and cell phone addiction has a significant positive effect on cell phone addiction.

**Table 5:** Results of the test of the Moderating role of psychological capital.

Research Pathways	Estimate	S.E.	C.R.	P
MC←SA	0.389	0.034	11.766	***
PC←SA	-0.256	0.033	-7.752	***
MC←SCC	0.189	0.024	6.486	***
SA←SCC	-0.093	0.024	-3.059	0.002
PC←SCC×SA	0.127	0.027	4.287	***
MC←SCC×SA	0.117	0.026	4.165	***

***p<0.000.

## 5 Discussion

### 5.1 Discussion of results

We designed a mediated moderation model to evaluate the consequences of college students’ SA on malicious inventiveness and investigated the mediating function of psychological capital in the association between SA and malevolent creativity among university students, together with the moderating effect of SCC on the correlations between SA and psychological capital, and between SA and malevolent creativity. The results confirmed the hypotheses and aligned with previous research.

Firstly, this study verified a substantial predictive correlation between SA and malevolent creativity (β=0.514, P<0.001), indicating that Hypothesis 1 is valid, consistent with previous studies (119, 120). Some studies have shown that smartphone addicts who are chronically dependent on their phones are prone to adverse emotions such as depression, anxiety, and loneliness, which may manifest themselves in unethical or malevolent creative behaviors when negative emotions continue to accumulate and are not properly regulated (116). Another explanation is that the frequent information stimulation and immediate feedback brought by cell phones can interfere with an individual’s attention, making it easier to be distracted by thinking and, thus, weakening self-control over behavior (121). Additionally, SA, similar to other addictive behaviors, alters the brain’s reward and punishment mechanisms, especially the dopamine system, making addicts more sensitive to instant gratification and novel stimuli (122). As a result, smartphone addicts with reduced levels of self-control are more inclined to engage in “off-the-wall” or “off-normal” thrill-seeking and risk-taking behaviors, and malevolent creativity may be a way to satisfy this need.

Secondly, the results of the study confirmed H2a and H2b. SA has a detrimental predictive impact on psychological capital, it aligns with the findings of previous research (123). Individuals glued to smartphones are subjected to an excessive volume of superfluous information for an extended period, which leads to the destruction and depletion of psychological resources, and they are prone to distraction and inefficiency regarding life and work challenges, affecting the accumulation of psychological capital such as resilience and self-confidence. Although no studies have directly examined the impact of psychological capital on malevolent creativity, some studies indirectly prove that psychological capital alleviates students’ antisocial problems and malevolent creativity. Individuals with psychological capital have greater self-regulation and effectively control impulses to deal with problems in a more rational and constructive manner (72, 124), which reduces malevolent creativity (51). Meanwhile, people with elevated levels of psychological capital experience more happy emotions, which reduce negative emotions such as depression and anxiety (125) and build good social relationships (126). According to the social exchange theory, positive social interactions can reduce hostility and conflict and lower the incidence of malevolent behavior (127).

Thirdly, H2 has been validated. Psychological capital partially facilitated the connection between SA and malevolent creativity. This discovery aligns with psychological and sociological studies; when a college student is addicted to a smartphone, they are susceptible to adverse emotions and unethical conduct when they lack sufficient resources and self-control to alleviate the detrimental impacts of SA (128–131). However, psychological capital can be a powerful psychological resource that mitigates the negative effects of SA (132, 133), which reduces the prevalence of malevolent behaviors. To put it differently, the more powerful an individual’s psychological capital, the greater their willingness to moderate negative emotions and resource depletion associated with SA by actively and creatively engaging with the stressors.

Fourthly, this study discovered that college students’ SCC positively moderates the association between SA and psychological capital, and the findings validate H3. According to the literature, college students possessing SCC are more inclined to identify and adapt to environmental stressors and challenges. Thus, they show higher self-regulatory abilities and coping strategies (134). Meanwhile, SCC is a psychological regulator. When individuals are confronted with behavioral problems, such as SA, those with SCC are more likely to be aware of the negative effects of addictive behaviors and are able to adopt effective strategies to manage and reduce the onset of addiction (135), which is widely considered to be a behavior similar in nature to addiction. Therefore, SCC can positively regulate SA and positively regulate psychological capital. SA is disturbed by SCC, serving as a moderating element, when it negatively affects psychological capital. Under the regulation of high SCC, the detrimental consequences of SA can be mitigated and potentially converted into good self-efficacy.

Fifthly, this study discovered that SCC negatively moderates the association between college students’ psychological capital and malevolent creativity. The greater the degree of SCC, the weaker the effect of college students’ psychological capital on malevolent creativity, which verifies Hypothesis H4. This discovery aligns with psychological and social research, which indicates that persons possessing a distinct self-concept have a comprehensive understanding and solid identity for both their essence and appropriate behavior. This understanding and identification allows them to clearly identify their goals and values when faced with decision making. They tend to choose solutions that are consistent with their own perceptions and social norms (136), and the malevolent creativity of this group of individuals is usually low.

In summary, SCC serves a dual moderating function in the association between SA and both psychological capital and malevolent creativity. It assists individuals in mitigating the adverse consequences of SA and improving their self-efficacy. University students maintain a high degree of SCC to counter the development of malevolent creativity by maintaining a low level of malevolent creativity even when their psychological capital is low.

### 5.2 Implications of the study

Regarding theoretical significance, this study correlates the prevalence of malevolent creativity with college students’ comprehension of the creative process, particularly in the context of contemporary SA rates. The analysis of the moderating and mediating effects suggests that college students’ self-clarity enhances their psychological capital and reduces the degree of malevolent creativity of the smartphone-addicted ones. From a pragmatic point of view, the relationship between SA, malevolent creativity, psychological capital, self-concept can assist school administrators effectively inhibit college students’ malevolent creativity, focus on their cell phone use and mental health, and help them use their cell phones rationally, and enhance their psychological capital. For example, teachers can facilitate opportunities for pupils to participate in significant activities while upholding elevated academic standards. Moreover, school administrators can establish diverse support systems for pupils that do not depend exclusively on external motivation or ideological and political instruction.

### 5.3 Limitations and future directions

This research has specific limitations and suggests pathways for additional research. First, although the primary objective of this study was to examine the effect of SA on university students’ malevolent creativity and its related mechanisms, the effects of variables such as age, gender, and major on this relationship were not controlled for owing to the constraints of data acquisition. Second, we employed a cross-sectional design. While cross-sectional studies coupled with data analysis can investigate the correlation among many variables, the causal relationship between these variables remains inadequately elucidated.

## 6 Conclusion

We developed a moderated mediation model to examine the effects of SA on college students’ malevolent creativity, and the research results revealed that (1) SA was a strong predictor of malevolent creativity (2); psychological capital partially mitigated the connection between SA and malevolent creativity; and (3) self-concept distinctly moderated the effect of SA. The results illuminate the mechanisms influencing the relationship between SA and harmful creativity in university students.

## Data Availability

The raw data supporting the conclusions of this article will be made available by the authors, without undue reservation.

## References

[1] Lepp A Barkley JE Karpinski AC. The relationship between cell phone use, academic performance, anxiety, and Satisfaction with Life in college students. Comput Hum Behav. (2014) 31:343–50. doi: 10.1016/j.chb.2013.10.049

[2] Holloway P Kenna T Linehan D O’Connor R Bradley H O’Mahony B. Active learning using a smartphone app: analysing land use patterns in Cork City, Ireland. J Geogr High Educ. (2021) 45:47–62. doi: 10.1080/03098265.2020.1802703

[3] Tiantian Y Razali AB Zulkifli NN Jeyaraj JJ. The effects of collaborative mobile learning approach on academic performance: The mediating role of social interaction, and learning motivation. JPR. (2024) 3:209–29. doi: 10.33902/JPR.202426264

[4] Wardani IS Munir. WA. The effect of smartphones media to improve critical thinking skills student of elementary school. jppipa pendidikan ipa fisika biologi kimia. (2024) 10:479–86. doi: 10.29303/jppipa.v10i2.3346

[5] Kvintova J Novotny JS Liu H Vachova L Kantor J. Path analysis reveals cross-country differences between Czech and Chinese university students in effect of internet and smartphone addiction, mental health, and personality traits on academic achievement in the post-pandemic era. BMC Psychol. (2024) 12:567. doi: 10.1186/s40359-024-02069-x 39420418 PMC11487747

[6] Chen L Yan Z Tang W Yang F Xie X He J. Mobile phone addiction levels and negative emotions among Chinese young adults: The mediating role of interpersonal problems. Comput Hum Behav. (2016) 55:856–66. doi: 10.1016/j.chb.2015.10.030

[7] Karaoglan Yilmaz FG Ustun AB Zhang K Yilmaz R. Smartphone addiction, nomophobia, depression, and social appearance anxiety among college students: a correlational study. J Rat-Emo Cognitive-Behav Ther. (2024) 42:305–21. doi: 10.1007/s10942-023-00516-z

[8] Kaya B. Smartphone addiction and psychological wellbeing among adolescents: the multiple mediating roles of academic procrastination and school burnout. Br J Guidance Counselling. (2024) 52:815–29. doi: 10.1080/03069885.2024.2304208

[9] Wei J Dang J Mi Y Zhou M. Mobile phone addiction and social anxiety among Chinese adolescents: Mediating role of interpersonal problems. psicol. (2024) 40:103–9. doi: 10.6018/analesps.381801

[10] Wang J Xu X Zuo L Wang H Yang G. Mobile phone addiction and insomnia among college students in China during the COVID-19 pandemic: a moderated mediation model. Front Public Health. (2024) 12:1338526. doi: 10.3389/fpubh.2024.1338526 38528859 PMC10961390

[11] Darabiyan P Nazari H Zarea K Ghanbari S Raiesifar Z Khafaie MA. Relationship between alexithymia and mobile phone addiction with an emphasis on the mediating role of anxiety, stress, and depression: a structural model analysis. Jundishapur J Chronic Dis Care. (2024) 13:85–95. doi: 10.5812/jjcdc-143458

[12] Organization WH. Public health implications of excessive use of the internet, computers, smartphones and similar electronic devices: meeting report, Main Meeting Hall, Foundation for Promotion of Cancer Research, National Cancer Research Centre, Tokyo, Japan, 27-29 August 2014 (2015). World Health Organization. Available online at: https://iris.who.int/handle/10665/184264 (Accessed January 10, 2025).

[13] Shang Z Wang D Liu Z Zhang X. Exploring the impact of smartphone addiction on mental health among college students during the COVID-19 pandemic: The role of resilience and parental attachment. J Affect Disord. (2024) 367:756–67. doi: 10.1016/j.jad.2024.09.035 39260581

[14] Li Y Zhang L Yang Y Xiang S Hu W. Addiction-prone personality and creative cognitive styles: a moderated mediation model of novelty seeking and depression tendency. Psychol Rep. (2024) 127:1214–36. doi: 10.1177/00332941221137239 36315897

[15] Guan J Yang Y Ma W Li G Liu C. The relationship between mobile phone use and creative ideation among college students: The roles of critical thinking and creative self-efficacy. In: Psychology of aesthetics, creativity, and the arts (2024). doi: 10.1037/aca0000695

[16] Cropley DH. Summary – the dark side of creativity: A differentiated model. In: Cropley DH Cropley AJ Kaufman JC Runco MA, editors. The dark side of creativity. Cambridge University Press (2010). p. 360–74. doi: 10.1017/CBO9780511761225.020

[17] Cropley DH Kaufman JC Cropley AJ. Malevolent creativity: A functional model of creativity in terrorism and crime. Creativity Res J. (2008) 20:105–15. doi: 10.1080/10400410802059424

[18] Fu H Zhang Z. The relationship between Honesty-Humility and malevolent creativity: sequential mediation models with prosocial moral emotional traits and prosocial tendencies. Curr Psychol. (2024) 43:7424–36. doi: 10.1007/s12144-023-04941-2

[19] Wang D Wang D Chen W. The relationship between adolescents&rsquo; resilience and their malevolent creative behaviors. Acta Psychol Sin. (2022) 54:154–67. doi: 10.3724/SP.J.1041.2022.00154

[20] Kim B Kim N Park KE. Effect of university students’ smartphone addiction on their life management: mediating effects of protective factor and risk factor. J Korea Contents Assoc. (2018) 18:594–606. doi: 10.5392/JKCA.2018.18.08.594

[21] Pickard H. Addiction and the self. Nous. (2021) 55:737–61. doi: 10.1111/nous.12328

[22] Kong F Lan N Zhang H Sun X Zhang Y. How does social anxiety affect Mobile phone dependence in adolescents? The mediating role of self-concept clarity and self-esteem. Curr Psychol. (2022) 41:8070–7. doi: 10.1007/s12144-020-01262-6

[23] Xiao Q-L Ding Y-Q Cao X-X Chen W-Y Lian S-L Zhu X-W. Mobile phone addiction and emptiness among Chinese college students: the chain mediating role of attention control and self-concept clarity. Curr Psychol. (2024) 43:25297–310. doi: 10.1007/s12144-024-06221-z

[24] Billieux J van der Linden M d’Acremont M Ceschi G Zermatten A. Does impulsivity relate to perceived dependence on and actual use of the mobile phone? Appl Cogn Psychol. (2007) 21:527–37. doi: 10.1002/acp.1289

[25] Liu Q Yang Y lin Y Yu S Zhou Z. Smartphone addiction: concepts, measurements, and factors. Chin J Clin Psychol. (2017) 25:82–7. doi: 10.16128/j.cnki.1005-3611.2017.01.019

[26] Gökçearslan Ş Mumcu FK Haşlaman T Çevik YD. Modelling smartphone addiction: the role of smartphone usage, self-regulation, general self-efficacy and cyberloafing in university students. Comput Hum Behav. (2016) 63:639–49. doi: 10.1016/j.chb.2016.05.091

[27] Rai S Saroshe S Khatri A Sirohi S Dixit S. A cross sectional study to assess the effects of excessive use of smartphones among professional college going students. Int J Community Med Public Health. (2016) 3(3):758–63. doi: 10.18203/2394-6040.ijcmph20160647

[28] Bianchi A Phillips JG. Psychological predictors of problem mobile phone use. CyberPsychology Behav. (2005) 8:39–51. doi: 10.1089/cpb.2005.8.39 15738692

[29] Billieux J Philippot P Schmid C Maurage P De Mol J van der Linden M. Is dysfunctional use of the mobile phone a behavioural addiction? Confronting symptom-based versus process-based approaches. Clin Psychol Psychother. (2015) 22:460–8. doi: 10.1002/cpp.1910 24947201

[30] Lee Y-K Chang C-T Lin Y Cheng Z-H. The dark side of smartphone usage: Psychological traits, compulsive behavior and technostress. Comput Hum Behav. (2014) 31:373–83. doi: 10.1016/j.chb.2013.10.047

[31] Billieux J Maurage P Lopez-Fernandez O Kuss DJ Griffiths MD. Can disordered mobile phone use be considered a behavioral addiction? An update on current evidence and a comprehensive model for future research. Curr Addict Rep. (2015) 2:156–62. doi: 10.1007/s40429-015-0054-y

[32] Mahapatra S. Smartphone addiction and associated consequences: role of loneliness and self-regulation. Behav Inf Technol. (2019) 38:833–44. doi: 10.1080/0144929X.2018.1560499

[33] Fabio RA Stracuzzi A Lo Faro R. Problematic smartphone use leads to behavioral and cognitive self-control deficits. IJERPH. (2022) 19:7445. doi: 10.3390/ijerph19127445 35742695 PMC9223448

[34] Dai C Tai Z Ni S. Smartphone use and psychological well-being among college students in China: a qualitative assessment. Front Psychol. (2021) 12:708970. doi: 10.3389/fpsyg.2021.708970 34566786 PMC8458628

[35] Kwon M Kim D-J Cho H Yang S. The smartphone addiction scale: development and validation of a short version for adolescents. PloS One. (2013) 8:e83558. doi: 10.1371/journal.pone.0083558 24391787 PMC3877074

[36] De-Sola Gutiérrez J Rodríguez de Fonseca F Rubio G. Cell-phone addiction: a review. Front Psychiatry. (2016) 7:175. doi: 10.3389/fpsyt.2016.00175 27822187 PMC5076301

[37] Ming Z Qin X Lingyi Z. Research progress in mobile phone dependence: causes, outcomes and interventions. Chin J Special Educ. (2019) 0:88–96. doi: 10.3969/j.issn.1007-3728.2019.11.015

[38] Soni R Upadhyay R Jain M. Prevalence of smart phone addiction, sleep quality and associated behaviour problems in adolescents. Int J Res Med Sci. (2017) 5:515. doi: 10.18203/2320-6012.ijrms20170142

[39] Habib U Ullah H Tariq Y. Awareness level of ocular symptoms caused by smart phone Use. Ophthalmology Update. (2019) 17(3):135.

[40] Parasuraman S Sam AT Yee SWK Chuon BLC Ren LY. Smartphone usage and increased risk of mobile phone addiction: A concurrent study. Int J Pharm Invest. (2017) 7:125–31. doi: 10.4103/jphi.JPHI_56_17 PMC5680647 29184824

[41] Hu H Yang X Mo PKH Zhao C Kuang B Zhang G. How mobile phone addiction is associated with suicidal ideation in university students in China: Roles of depression and online social support. Front Psychol. (2022) 13:1001280. doi: 10.3389/fpsyg.2022.1001280 36619077 PMC9816797

[42] Shinetsetseg O Jung YH Park YS Park E-C Jang S-Y. Association between smartphone addiction and suicide. IJERPH. (2022) 19:11600. doi: 10.3390/ijerph191811600 36141872 PMC9517102

[43] Choi M-Y Ji-Soo K. Influence of cell phone addiction on communication skills and interpersonal relationship ability of adolescents. J Korean Soc School Health. (2016) 29:149–55. doi: 10.15434/KSSH.2016.29.3.149

[44] Shahroudi S Soltani F Nouri N Rigi A. The relationship between cell-phone addiction with the academic achievement motivation and academic performance of students in Khash Baluchestan. Shenakht J Psychol Psychiatry. (2019) 5:57–70. doi: 10.29252/shenakht.5.6.57

[45] Gvion Y Apter A. Aggression, impulsivity, and suicide behavior: A review of the literature. Arch Suicide Res. (2011) 15:93–112. doi: 10.1080/13811118.2011.565265 21541857

[46] El-Murad J West DC. The definition and measurement of creativity: what do we Know? J Adv Res. (2004) 44:188–201. doi: 10.1017/S0021849904040097

[47] Ryhammar L Brolin C. Creativity Research: historical considerations and main lines of development. Scandinavian J Educ Res. (1999) 43:259–73. doi: 10.1080/0031383990430303

[48] Amabile TM. The meaning and measurement of creativity. In: The social psychology of creativity. Springer New York, New York, NY (1983). p. 17–35. doi: 10.1007/978-1-4612-5533-8_2

[49] Runco MA Jaeger GJ. The standard definition of creativity. Creativity Res J. (2012) 24:92–6. doi: 10.1080/10400419.2012.650092

[50] Plucker JA Beghetto RA Dow GT. Why isn’t creativity more important to educational psychologists? Potentials, pitfalls, and future directions in creativity research. Educ Psychol. (2004) 39:83–96. doi: 10.1207/s15326985ep3902_1

[51] Harris DJ Reiter-Palmon R Kaufman JC. The effect of emotional intelligence and task type on malevolent creativity. Psychol Aesthetics Creativity Arts. (2013) 7:237–44. doi: 10.1037/a0032139

[52] Amabile TM. A model of creativity and innovation in organizations, in: Research in organizational behavior (1988). Available online at: https://www.semanticscholar.org/paper/A-Model-of-Creativity-and-Innovation-in-Amabile/2c1b89386317ad2e1f491c566875f574e64b2043 (Accessed January 10, 2025).

[53] Sekowski A. Creativity in context: update to the social psychology of creativity. High Ability Stud. (1999) 10:233. doi: 10.4324/9780429501234

[54] Sternberg RJ Lubart TI. An investment theory of creativity and its development. Hum Dev. (1991) 34:1–31. doi: 10.1159/000277029

[55] Kaufman JC Beghetto RA. Beyond big and little: the four C model of creativity. Rev Gen Psychol. (2009) 13:1–12. doi: 10.1037/a0013688

[56] Glaveanu V Lubart T Bonnardel N Botella M Biaisi P-MD Desainte-Catherine M. Creativity as action: findings from five creative domains. Front Psychol. (2013) 4:176. doi: 10.3389/fpsyg.2013.00176 23596431 PMC3627136

[57] Rhodes M. An analysis of creativity. Phi Delta Kappan. (1961) 42:305–10. doi: 10.2307/20342603

[58] Muris P Merckelbach H Otgaar H Meijer E. The malevolent side of human nature: a meta-analysis and critical review of the literature on the dark triad (Narcissism, Machiavellianism, and Psychopathy). Perspect Psychol Sci. (2017) 12:183–204. doi: 10.1177/1745691616666070 28346115

[59] Morales Rodríguez FM Lozano JMG Linares Mingorance P Pérez-Mármol JM. Influence of smartphone Use on emotional, cognitive and educational dimensions in university students. Sustainability. (2020) 12:6646. doi: 10.3390/su12166646

[60] Geng Y Shi Y Hu W Jin W Zhang Y Zhan T. Fight Injustice with darkness: The effect of early life adversity on malevolent creativity Behavior. J Creative Behav. (2024) 58:279–96. doi: 10.1002/jocb.648

[61] Perchtold-Stefan CM Rominger C Papousek I Fink A. Antisocial schizotypy is linked to malevolent creativity. Creativity Res J. (2022) 34:355–67. doi: 10.1080/10400419.2021.2012633

[62] Cheng R Lu K Hao N. The effect of anger on malevolent creativity and strategies for its emotion regulation. Acta Psychologica Sin. (2021) 53:847. doi: 10.3724/SP.J.1041.2021.00847

[63] Roberts JA Pullig C Manolis C. I need my smartphone: A hierarchical model of personality and cell-phone addiction. Pers Individ Dif. (2015) 79:13–9. doi: 10.1016/j.paid.2015.01.049

[64] James C Weinstein E Mendoza K. Teaching digital citizens in today’s world. In: *Research and insights behind the Common Sense K–12 Digital Citizenship Curriculum*,. Common Sense Media, San Francisco, CA (2021).

[65] Jiaojiao LI Bibo XU Hailong YUAN Xiyang. The relationship between social exclusion YIN. and malevolent creativity of college students: the chain mediating effect of coping styles and aggressiveness. psychol Dev Educ. (2024) 40:667–74. doi: 10.16187/j.cnki.issn1001-4918.2024.05.07

[66] Kim I-K Park S-W Choi H-M. The relationship among smartphone addiction, communication ability, loneliness and interpersonal relationship for university students. J Korea Academia-Industrial cooperation Soc. (2017) 18:637–48. doi: 10.5762/KAIS.2017.18.1.637

[67] Volkmer SA Lermer E. Unhappy and addicted to your phone?—Higher mobile phone use is associated with lower well-being. Comput Hum Behav. (2019) 93:210–8. doi: 10.1016/j.chb.2018.12.015

[68] Seki T Hamazaki K Natori T Inadera H. Relationship between internet addiction and depression among Japanese university students. J Affect Disord. (2019) 256:668–72. doi: 10.1016/j.jad.2019.06.055 31299448

[69] Yi O Yehua W Huimin Z. The relationship between college students’ positive psychological quality and psychological resilience: parallel mediating roles of social support and mobile phone dependence. Adv Psychol. (2023) 13:6245. doi: 10.12677/AP.2023.1312797

[70] Goldsmith AH Veum JR Darity W. The impact of PSYCHOLOGICAL and human capital on wages. Economic Inq. (1997) 35:815–29. doi: 10.1111/j.1465-7295.1997.tb01966.x

[71] Luthans F Luthans KW Luthans BC. Positive psychological capital: beyond human and social capital. Business Horizons. (2004) 47:45–50. doi: 10.1016/j.bushor.2003.11.007

[72] Luthans F Avolio BJ Avey JB Norman SM. Positive psychological capital: Measurement and relationship with performance and satisfaction. Personnel Psychol. (2007) 60:541–72. doi: 10.1111/j.1744-6570.2007.00083.x

[73] Avey JB Reichard RJ Luthans F Mhatre KH. Meta-analysis of the impact of positive psychological capital on employee attitudes, behaviors, and performance. Hum Resource Dev Q. (2011) 22:127–52. doi: 10.1002/hrdq.20070

[74] Letcher L. Psychological capital and wages: a behavioral economic approach (2003). United States – Kansas: Kansas State University. Available online at: https://www.proquest.com/docview/305317629/abstract/D42ED837F6C24899PQ/1 (Accessed January 10, 2025).

[75] Avolio BJ Bass BM Jung DI. Re-examining the components of transformational and transactional leadership using the multifactor leadership. J Occupat Organ Psyc. (1999) 72:441–62. doi: 10.1348/096317999166789

[76] Luthans F Youssef-Morgan CM. Psychological capital: an evidence-based positive approach. Annu Rev Organ Psychol Organ Behav. (2017) 4:339–66. doi: 10.1146/annurev-orgpsych-032516-113324

[77] Dong Y Xiaoxuan W Wenhui F. Effects of physical exercise on school bullying among junior high school students: chain-mediated effects of psychological capital and self-control. Chin J Health Psychol. (2023) 31:733–39. doi: 10.13342/j.cnki.cjhp.2023.05.018

[78] Kuo Z Sai Z Yinghong D. Positive psychological capital:measurement and relationship with mental health. Stud Psychol Behav. (2010) 8:58. doi: 10.3969/j.issn.1672-0628.2010.01.011

[79] Bin L Hongyu M Yongyu G. The retrospect and prospect of the mechanism of psychological capital. psychol Res. (2014) 7:53–63. doi: 10.3969/j.issn.2095-1159.2014.06.009

[80] Xiangyang B. Psychological capital, emotional adaptation, and internet addiction in college students-a full effects moderation model analysis based on gender differences. Youth Stud. (2017) 3:42–52. doi: CNKI:SUN:QNYJ.0.2017-03-005

[81] Yilmaz R Karaoglan Yilmaz FG. Problematic internet use in adults: the role of happiness, psychological resilience, dispositional hope, and self-control and self-management. J Rat-Emo Cognitive-Behav Ther. (2023) 41:727–45. doi: 10.1007/s10942-022-00482-y PMC9548420 36247047

[82] Chen H Wang C Lu T Tao B Gao Y Yan J. The relationship between physical activity and college students’ mobile phone addiction: the chain-based mediating role of psychological capital and social adaptation. IJERPH. (2022) 19:9286. doi: 10.3390/ijerph19159286 35954644 PMC9367822

[83] Wang W Mehmood A Li P Yang Z Niu J Chu H. Perceived stress and smartphone addiction in medical college students: the mediating role of negative emotions and the moderating role of psychological capital. Front Psychol. (2021) 12:660234. doi: 10.3389/fpsyg.2021.660234 34366978 PMC8336678

[84] Chen S Wu Q. The study on the relationship between psychological capital and young teachers’ career identity: the mediating role of job involvement. Univ Educ Sci. (2018) 10:59–68. doi: 10.3969/j.issn.1672-0717.2018.01.011

[85] China Journal of Health Psychology Mou Z. The current situation of cell phone addiction among college students in local colleges and universities in borderland ethnic areas. China J Health Psychol. (2017) 25:1400–3. doi: 10.13342/j.cnki.cjhp.2017.09.032

[86] Li XX Wang MF Feng XJ He LL Liang J. The influence of stress perception on mobile phone addiction tendency in nursing undergraduates: the mediating role of self-control and the moderating role of psychological capital. (2024) 1–18. Available online at: https://assets-eu.researchsquare.com/files/rs-3827283/v1/136dd8d1-e043-434b-b54d-a3e40e32db7c.pdf?c=1704479993. 10.1186/s12912-025-03753-y PMC12379332 40855430

[87] Ullah I Hameed RM Mahmood A. The impact of proactive personality and psychological capital on innovative work behavior: evidence from software houses of Pakistan. EJIM. (2024) 27:1967–85. doi: 10.1108/EJIM-01-2022-0022

[88] Ghafoor A Haar J. Does job stress enhance employee creativity? Exploring the role of psychological capital. PR. (2022) 51:644–61. doi: 10.1108/PR-08-2019-0443

[89] Szabó E Körmendi A Kurucz G Cropley D Olajos T Pataky N. Personality traits as predictors of malevolent creative ideation in offenders. Behav Sci (Basel). (2022) 12:242. doi: 10.3390/bs12070242 35877312 PMC9311653

[90] Araban M Montazeri A Stein LAR Karimy M Mehrizi AAH. Prevalence and factors associated with disruptive behavior among Iranian students during 2015: a cross-sectional study. Ital J Pediatr. (2020) 46:85. doi: 10.1186/s13052-020-00848-x 32552890 PMC7301520

[91] Meng X Yiduo Y. The concept,measurement,influencing factors and effects of psychological capital. J East China Normal University: Educ Sci. (2014) 32:84–92. doi: 10.3969/j.issn.1000-5560.2014.03.012

[92] Simsek E Balaban Sali J. The role of internet addiction and social media membership on university students’ psychological capital. Contemp Educ Technol. (2014) 5:1–20. doi: 10.30935/cedtech/6127

[93] Lu H Xie C Lian P Yu C Xie Y. Psychosocial factors predict the level of aggression of people with drug addiction: a machine learning approach. Psychol Health Med. (2022) 27:1168–75. doi: 10.1080/13548506.2021.1910321 33874841

[94] Binyu W Zhe G. The effect of aggression on malevolent creativity:Analysis of chain mediating effect. psychol Res. (2021) 14:558–64. doi: 10.3969/j.issn.2095-1159.2021.06

[95] Campbell JD Trapnell PD Heine SJ Katz IM Lavallee LF Lehman DR. Self-concept clarity: Measurement, personality correlates, and cultural boundaries. J Pers Soc Psychol. (1996) 70:141–56. doi: 10.1037/0022-3514.70.1.141

[96] Noyman-Veksler G Weinberg D Fennig S Davidson L Shahar G. Perceived stigma exposure in schizophrenia: the key role of self-concept clarity. Self Identity. (2013) 12:663–74. doi: 10.1080/15298868.2012.732265

[97] Shin JY Steger MF Henry KL. Self-concept clarity’s role in meaning in life among American college students: A latent growth approach. Self Identity. (2016) 15:206–23. doi: 10.1080/15298868.2015.1111844

[98] Ritchie TD Sedikides C Wildschut T Arndt J Gidron Y. Self-concept clarity mediates the relation between stress and subjective well-being. Self Identity. (2011) 10:493–508. doi: 10.1080/15298868.2010.493066

[99] Alessandri G De Longis E Golfieri F Crocetti E. Can self-concept clarity protect against a pandemic? A daily study on self-concept clarity and negative affect during the COVID-19 outbreak. Identity. (2021) 21:6–19. doi: 10.1080/15283488.2020.1846538

[100] jie L xiang Z Chunjiang F. The effect of positive thinking on cell phone dependence in middle school students - the chain mediating role of social anxiety and self-concept clarity. Educ Res Monthly. (2022) 8:70–6. doi: 10.16477/j.cnki.issn1674-2311.2022.08.012

[101] Fang L Qin-yao W Lin-peng Z Xue Z Bi-yun W. Self-concept clarity and smartphone addiction among college students: the mediating effect of self-esteem and social anxiety. Chin J Clin Psychol. (2019) 27:900–4. doi: 10.16128/j.cnki.1005-3611.2019.05.009

[102] Mitchell KS Reiter-Palmon R. Chapter 4 - Malevolent creativity: personality, process, and the larger creativity field. In: Kapoor H Kaufman JC, editors. *Creativity and morality.* Explorations in creativity research. Academic Press (2023). p. 47–68. doi: 10.1016/B978-0-323-85667-6.00004-9

[103] Gavidia-Payne S Denny B Davis K Francis A Jackson M. Children’s self-concept: parental school engagement and student–teacher relationships in rural and urban Australia. Soc Psychol Educ. (2015) 18:121–36. doi: 10.1007/s11218-014-9277-3

[104] Feinstein BA Davila J Yoneda A. Self-concept and self-stigma in lesbians and gay men. Psychol Sexuality. (2012) 3:161–77. doi: 10.1080/19419899.2011.592543

[105] Campbell JD Fehr B. Self-esteem and perceptions of conveyed impressions: Is negative affectivity associated with greater realism? J Pers Soc Psychol. (1990) 58:122–33. doi: 10.1037/0022-3514.58.1.122 2308069

[106] Zhang J Zheng S Hu Z. The effect of physical exercise on depression in college students: the chain mediating role of self-concept and social support. Front Psychol. (2022) 13:841160. doi: 10.3389/fpsyg.2022.841160 35651580 PMC9150849

[107] Bechtoldt MN De Dreu CKW Nijstad BA Zapf D. Self-concept clarity and the management of social conflict. J Pers. (2010) 78:539–74. doi: 10.1111/j.1467-6494.2010.00626.x 20433630

[108] Hertel AW Sokolovsky AS Mermelstein RJ. The relationship of self-concept clarity with perceived stress, general anxiety, and depression among young adults. J Soc Clin Psychol. (2024) 43:473–91. doi: 10.1521/jscp.2024.43.5.473

[109] Hyde LW Shaw DS Moilanen KL. Developmental precursors of moral disengagement and the role of moral disengagement in the development of antisocial behavior. J Abnorm Child Psychol. (2010) 38:197–209. doi: 10.1007/s10802-009-9358-5 19777337 PMC2858331

[110] Heym N Firth J Kibowski F Sumich A Egan V Bloxsom CAJ. Empathy at the heart of darkness: empathy deficits that bind the dark triad and those that mediate indirect relational aggression. Front Psychiatry. (2019) 10:95. doi: 10.3389/fpsyt.2019.00095 30930800 PMC6423894

[111] Tian S Sun G. Relationship between self-concept clarity, mental toughness, athlete engagement, and athlete burnout in swimmers during and after the COVID-19 pandemic. Int J Sport Exercise Psychol. (2024) 22:1401–18. doi: 10.1080/1612197X.2023.2224824

[112] Xiang G Li Q Du X Liu X Xiao M Chen H. Links between family cohesion and subjective well-being in adolescents and early adults: The mediating role of self-concept clarity and hope. Curr Psychol. (2022) 41:76–85. doi: 10.1007/s12144-020-00795-0

[113] Burns RA Crisp DA Burns RB. Re-examining the reciprocal effects model of self-concept, self-efficacy, and academic achievement in a comparison of the Cross-Lagged Panel and Random-Intercept Cross-Lagged Panel frameworks. Br J Educ Psychol. (2020) 90:77–91. doi: 10.1111/bjep.12265 30657590

[114] Kline RB. *Principles and practice of structural equation modeling.* Fifth edition. New York: The Guilford Press (2023). p. 494.

[115] Leung L. Linking psychological attributes to addiction and improper use of the mobile phone among adolescents in hong kong. J Children Media. (2008) 2:93–113. doi: 10.1080/17482790802078565

[116] Hao N Tang M Yang J Wang Q Runco MA. A new tool to measure malevolent creativity: the malevolent creativity behavior scale. Front Psychol. (2016) 7:682. doi: 10.3389/fpsyg.2016.00682 27242596 PMC4870273

[117] Podsakoff PM MacKenzie SB Lee J-Y Podsakoff NP. Common method biases in behavioral research: A critical review of the literature and recommended remedies. J Appl Psychol. (2003) 88:879–903. doi: 10.1037/0021-9010.88.5.879 14516251

[118] Hayes AF. Beyond baron and kenny: statistical mediation analysis in the new millennium. Communication Monogr. (2009) 76:408–20. doi: 10.1080/03637750903310360

[119] Balta S Jonason P Denes A Emirtekin E Tosuntaş ŞB Kircaburun K. Dark personality traits and problematic smartphone use: The mediating role of fearful attachment. Pers Individ Dif. (2019) 149:214–9. doi: 10.1016/j.paid.2019.06.005

[120] Servidio R Griffiths MD Demetrovics Z. Dark triad of personality and problematic smartphone use: a preliminary study on the mediating role of fear of missing out. IJERPH. (2021) 18:8463. doi: 10.3390/ijerph18168463 34444212 PMC8391539

[121] Al-Amri A Abdulaziz S Bashir S Ahsan M Abualait T. Effects of smartphone addiction on cognitive function and physical activity in middle-school children: a cross-sectional study. Front Psychol. (2023) 14:1182749. doi: 10.3389/fpsyg.2023.1182749 37645064 PMC10461096

[122] Koob GF Volkow ND. Neurocircuitry of addiction. Neuropsychopharmacol. (2010) 35:217–38. doi: 10.1038/npp.2009.110 PMC2805560 19710631

[123] Zhang C Li G Fan Z Tang X Zhang F. Psychological capital mediates the relationship between problematic smartphone use and learning burnout in Chinese medical undergraduates and postgraduates: a cross-sectional study. Front Psychol. (2021) 12:600352. doi: 10.3389/fpsyg.2021.600352 34054634 PMC8155251

[124] Emami Khotbesara Z Mahdian H Bakhshipour A. Comparing the effectiveness of academic buoyancy and psychological capital training on academic procrastination in female high school students. ijes. (2024) 3:149–60. doi: 10.61838/kman.ijes.7.3.18

[125] Song R Sun N Song X. The efficacy of psychological capital intervention (PCI) for depression from the perspective of positive psychology: a pilot study. Front Psychol. (2019) 10:1816. doi: 10.3389/fpsyg.2019.01816 31447745 PMC6692487

[126] Slatten T Mutonyi BR Lien G. The impact of individual creativity, psychological capital, and leadership autonomy support on hospital employees’ innovative behaviour. BMC Health Serv Res. (2020) 20:1096. doi: 10.1186/s12913-020-05954-4 33246454 PMC7691957

[127] De Clercq D Belausteguigoitia I. How social interaction can prevent interpersonal conflict from inducing turnover intentions and diminishing championing behaviour. IJOA. (2023) 31:3582–602. doi: 10.1108/IJOA-07-2022-3350

[128] Masood A Feng Y Rasheed MI Ali A Gong M. Smartphone-based social networking sites and intention to quit: self-regulatory perspective. Behav Inf Technol. (2021) 40:1055–71. doi: 10.1080/0144929X.2020.1740787

[129] Schmidt-Barad T Chernyak-Hai L. Phubbing makes the heart grow callous: effects of phubbing on pro-social behavioral intentions, empathy and self-control. Psychol Rep. (2024), 332941241284917. doi: 10.1177/00332941241284917 39305235

[130] Shahzalal M Adnan HM. Attitude, self-control, and prosocial norm to predict intention to use social media responsibly: from scale to model fit towards a modified theory of planned behavior. Sustainability. (2022) 14:9822. doi: 10.3390/su14169822

[131] Swiatek AH Szcześniak M Aleksandrowicz B Zaczkowska D Wawer W Ścisłowska M. Problematic smartphone use and social media fatigue: the mediating role of self-control. Psychol Res Behav Manag. (2023) 16:211–22. doi: 10.2147/PRBM.S389806 PMC9884050 36718180

[132] Jiang Y. Problematic social media usage and anxiety among university students during the COVID-19 pandemic: the mediating role of psychological capital and the moderating role of academic burnout. Front Psychol. (2021) 12:612007. doi: 10.3389/fpsyg.2021.612007 33613391 PMC7892454

[133] Teymorzadeh H Mohammadipour M Bakhshipour A. Modeling the structural relationships of internet addiction based on academic self - regulation with the mediating role of academic resilience. . Inf Communication Technol Educ Sci. (2022) 12:103–22. Available online at: https://www.magiran.com/p2405219.

[134] Yang W Hu D Guo Y. The relationship between school bullying victimization and social mindfulness in middle school students: a chain mediating model of self-concept clarity and cognition reappraisal. Front Psychol. (2024) 15:1388301. doi: 10.3389/fpsyg.2024.1388301 39161691 PMC11330770

[135] Chen M Zeng X Chen Y. Self-concept and abstinence motivation in male drug addicts: coping style as a mediator. Soc Behav Pers. (2020) 48:1–15. doi: 10.2224/sbp.9334

[136] Cooper D Thatcher SMB. Identification in organizations: The role of self-concept orientations and identification motives. AMR. (2010) 35:516–38. doi: 10.5465/amr.35.4.zok516

